# Lower Plasma miR-223 Level Is Associated with Clopidogrel Resistance in Acute Coronary Syndrome: A Systematic Review and Meta-Analysis

**DOI:** 10.1155/2023/9322188

**Published:** 2023-08-17

**Authors:** Hang Cheng, Min Yang, Junli Hao, Kejie Chen, Quandan Tan, Song He, Fengkai Mao, Ming Yang, Yapeng Lin, Jie Yang

**Affiliations:** ^1^Department of Neurology, The First Affiliated Hospital of Chengdu Medical College, Chengdu, China; ^2^School of Bioscience and Technology, Cengdu Medical College, Chengdu, China; ^3^School of Public Health, Cengdu Medical College, Chengdu, China; ^4^International Clinical Research Center, Cengdu Medical College, Chengdu, China; ^5^Department of Neurology, Sichuan Provincial People's Hospital, University of Electronic Science and Technology of China, Chengdu, China

## Abstract

**Objectives:**

To evaluate the relationship between the plasma miR-223 expression level and clopidogrel resistance in acute coronary syndrome (ACS) patients.

**Methods:**

We performed a search for publications using online databases including PubMed, EMBASE, Cochrane Library, and Chinese Databases (CNKI database, Weipu database, and Wanfang database) from the inception of the databases to June 18, 2023, to identify studies reporting the relationship between the plasma miR-223 level and clopidogrel resistance in ACS patients. Two researchers independently searched and screened to ensure the consistency of the results and assess the quality of the included studies according to the Newcastle-Ottawa scale. A fixed-effects model was used for pooling data with STATA 14.0.

**Results:**

Four articles including 399 Chinese ACS patients were eligible for the meta-analysis. Low plasma miR-223 levels were independently correlated with clopidogrel resistance in Chinese ACS patients (OR 0.58, 95% CI: 0.33–1.04).

**Conclusion:**

Lower plasma miR-223 levels are associated with clopidogrel resistance in Chinese ACS patients, suggesting that miR-223 may be a potential diagnostic biomarker of clopidogrel resistance.

## 1. Introduction

Inhibition of platelet function by means of dual antiplatelet therapy (DAPT) is the cornerstone of treatment in acute coronary syndrome (ACS). For ACS patients, DAPT with aspirin and clopidogrel is the mainstay of treatment [1–3]. Several clinical trials and basic research studies have suggested that there is wide variability in the antiplatelet response in ACS patients receiving loading doses or a standard regimen of clopidogrel [4–9]. The risk of ischemic events is still high in ACS patients, which could be partially contributed by “clopidogrel resistance.” Clopidogrel resistance is usually defined as ADP-induced platelet aggregation function (PAF) exceeding 208 units, which often leads to new onset and recurrence of ischemic events [4]. Many factors, including expression of P2Y12 receptors on the platelet, pharmaceutical metabolic enzyme activity in the liver, drug-drug interaction, and demographic characteristics, are important risk factors for clopidogrel resistance [10].

MicroRNA is a small nonencoding RNA molecule that adjusts the functionality of multiple specific mRNA by adjusting translation and degradation of mRNA. It is estimated that more than 40% of the plasma microRNAs are derived from platelets [11]. Previous studies showed that microRNA-223 (miR-223) downregulates the expression of P2Y12 receptors on the platelet, which is the key protein for clopidogrel binding and PAF [12]. Moreover, small clinical samples suggest that the relationship between the plasma miR-223 level and clopidogrel resistance remains controversial [4–9]. Therefore, we explored the relationship between the plasma miR-223 level and clopidogrel resistance in ACS patients through a systematic review and meta-analysis.

## 2. Materials and Methods

### 2.1. Search Strategy

We performed a comprehensive search for publications using online English databases (including PubMed, EMBASE, and Cochrane Library) and Chinese database (including CNKI database, Weipu database, and Wanfang database) from the inception of the databases to Jun 18, 2023. The literature retrieval strategy was created by one author and then approved by the other authors, using keywords of “miR-223 OR microRNA-223 OR miRNA OR microRNA” AND “clopidogrel.” Moreover, all included studies were screened manually to identify further qualifying studies.

### 2.2. Study Inclusion and Exclusion Criteria

The following articles were included in our meta-analysis: (i) all adult participants received a diagnosis of ACS and received a loading dose of 300 mg aspirin plus 300 mg clopidogrel for at least 24 h, or 100 mg aspirin plus 75 mg clopidogrel for at least 5 days, to reach a stable drug concentration, (ii) all participants were separated into cohorts based on plasma miR-223 levels, and (iii) the odds ratio (OR) and 95% confidence intervals (CI) were available. Studies were excluded if they met the following criteria: (i) case reports, reviews, conference abstracts, letters, and animal studies, (ii) duplicated studies, and (iii) insufficient data required.

### 2.3. Data Extraction and Quality Assessment

All studies and data were independently reviewed and extracted by two authors (H.C. and M.Y.). A third author (J.Y.) was consulted for resolution of any conflicts. The Preferred Reporting Items for Systematic Reviews and Meta-Analyses (PRISMA) statement was followed for this systematic review [13]. Relevant data included authors, publication year, research location, recruitment duration, sample size, detection methods of the plasma miR-223 level, and PAF. There was no preregistration for the review. Study quality was evaluated according to the Newcastle-Ottawa Quality Assessment Scale (NOS) [14].

### 2.4. Statistical Analysis

The relationship between plasma miR-223 levels and clopidogrel resistance was examined by pooling the OR and 95% CI. A funnel plot and Egger's linear regression test to examine the publication bias. Statistical analyses were conducted in STATA 14.0 (StataCorp, TX, USA).

## 3. Results

### 3.1. Results of Literature Search and Characteristics of the Studies Included

**Figure 1:**
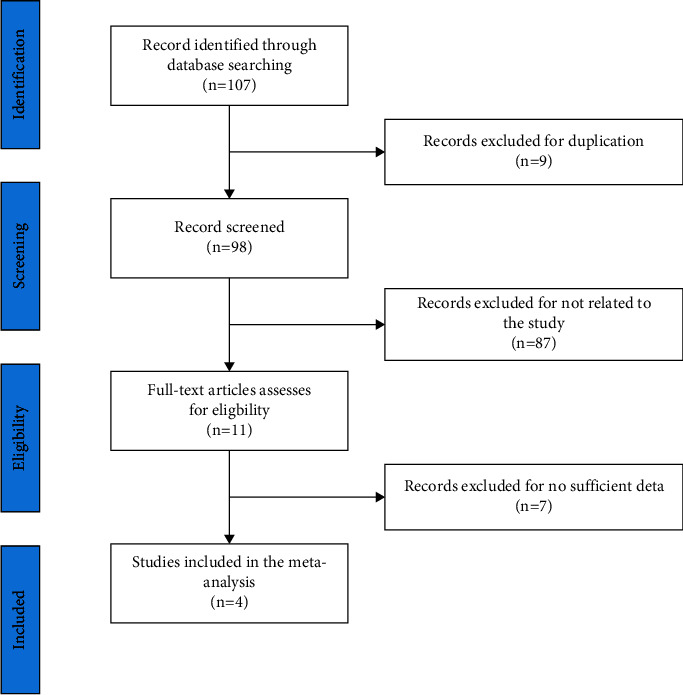
Flowchart showing the study selection process.

The study screen flowchart is summarized in Figure 1. We found 107 relevant articles in the initial search. After reviewing the titles and abstracts, 98 of them were deemed ineligible since they were duplicated articles, review articles, letters, or animal studies, etc. Then, we screened the remaining 11 studies for eligibility, of which 7 were further excluded for insufficient data. Finally, 4 articles including 399 patients were included in the meta-analysis.

All studies were conducted in China. Plasma miR-223 levels were examined via quantitative real-time polymerase chain reaction (qRT-PCR) in all studies, and clopidogrel and antiplatelet responsiveness were evaluated according to the calculation of the percentage of platelet aggregation. The NOS assessment score of the 4 included studies varies from 7 to 9, indicating that all 4 studies have high quality based on the quality assessment. More detailed information is presented in Table 1.

### 3.2. Relationship between the Plasma miR-223 Level and Clopidogrel Resistance

**Figure 2:**
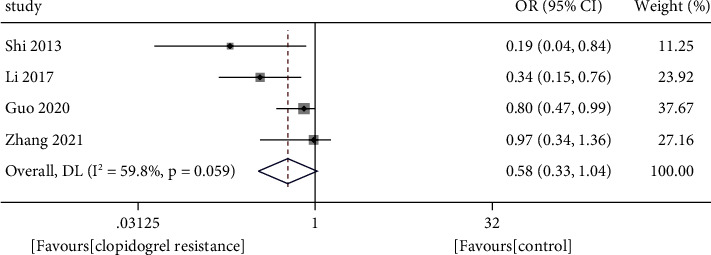
Forest plots showing odds ratio and 95% confidence intervals of clopidogrel resistance in acute coronary for the lower plasma miR-223 level.

**Figure 3:**
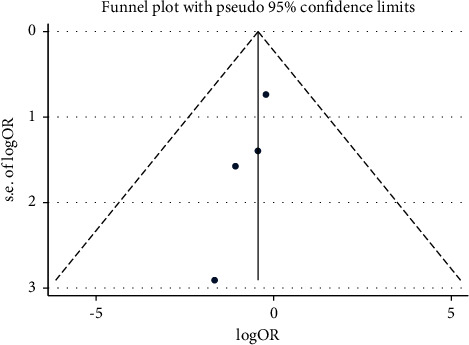
Funnel plot.

Based on pooling analysis, low plasma miR-223 levels were correlated with clopidogrel resistance in ACS patients (OR 0.58, 95% CI: 0.33–1.04, as shown in Figure 2). There was significant heterogeneity across studies (I2 = 59.8%; *P*=0.059). Evidence of publication bias was observed by the symmetrical funnel plot (shown in Figure 3) and Egger's linear regression test (*P*=0.021).

## 4. Discussion

Clopidogrel resistance is one of the key factors resulting in failure of clopidogrel antiplatelet therapy for ACS patients, and it is time-consuming and unstable to diagnose clopidogrel resistance by PAF [15]. Therefore, it is important to find easily detectable diagnostic biomarkers of clopidogrel resistance.

It is known that miR-223 levels are largely modulated during ACS events [16], and multiple studies have suggested that miR-223 can be exploited as a biomarker for platelet activation and that its levels are associated with antiplatelet treatment and the individual response to it [17–19]. Our meta-analysis shows that plasma miR-223 may be a diagnostic biomarker to predict clopidogrel resistance in patients with ACS. To the best of our knowledge, this is the first meta-analysis which indicated the clinical diagnostic role of miR-223 on clopidogrel resistance in ACS.

The mechanism of the lower plasma miR-223 level indicating clopidogrel resistance could be related to miR-223's regulation of the expression of the platelet P2Y12 receptor. The P2Y12 receptor is the key protein of platelet aggregation, and more expression of the platelet P2Y12 receptor leads to higher PAF. Schubert and Devine suggested that there is a miR-223 binding site in the 3 UTR of P2Y12 mRNA, miR-223 binds to the P2Y12 mRNA, and it reduces the expression of platelet P2Y12 receptor [20]. Recent studies have shown that miR-223 can regulate the expression of platelet P2Y12 receptors via posttranslational regulation, which might explain why lower levels of plasma miR-223 could increase platelet P2Y12 receptors and induce clopidogrel resistance [21]. However, Carino found that switching from a dual antiplatelet therapy including aspirin and clopidogrel to the more potent ticagrelor resulted in a decrease in plasma miR-233 levels, suggesting that the use of ticagrelor also decreased plasma miR-233 expression levels and induced ticagrelor resistance, which needs to be confirmed by further research [22].

There are several limitations to this meta-analysis. First, all studies were conducted in China, so the result may be not generalizable to other races. Second, the result was based on only 399 ACS patients from four articles and should be verified by large sample studies in the future. Third, the relationship between the plasma miR-223 level and the clinical outcome was not evaluated, which should be explored in the future studies. Fourth, the result of publication bias based on Egger's test may be unreliable. Finally, the cut-off value for identifying patients who are low responders to clopidogrel therapy varies.

All in all, our meta-analysis shows that lower plasma miR-223 levels are associated with clopidogrel resistance in Chinese ACS patients and that miR-223 may be a potential diagnostic biomarker of clopidogrel resistance. However, the current evidence level of most studies is relatively low for a small number of samples.

## Figures and Tables

**Table 1:** Characteristics of the studies included.

Study	Region	Recruitment time	Study type	Disease type	Sample size	Experimental sample	Analysis method	Platelet function test	miRNA detection method	Quality score
Shi et al. 2013	China	NS	Cohort study	NSTE-ACS	33	Plasma	Univariate/multivariate analysis	LTA	qRT-PCR	8
Li et al. 2017	China	2013-2014	Cohort study	NSTE-ACS	39	Plasma	Univariate/multivariate analysis	LTA	qRT-PCR	8
Guo et al. 2020	China	2017-2018	Cohort study	CHD	119	Plasma	Univariate/multivariate analysis	TEG	qRT-PCR	7
Zhang et al. 2021	China	2016-2017	Cohort study	NSTE-ACS	208	Plasma	Univariate/multivariate analysis	Flow cytometer	qRT-PCR	9

NS: not sure; NSTE-ACS: non-st-segment elevation acute coronary syndrome; LTA: light transmittance aggregometry; TEG: thrombelastograph coagulation analyzer; CHD: coronary heart disease.

## Data Availability

The data used to support the findings of this study are available from the corresponding author upon request.
